# Drivers and barriers to workplace-based HIV self-testing among high-risk men in Uganda: a qualitative study

**DOI:** 10.1186/s12889-021-11041-y

**Published:** 2021-05-27

**Authors:** Patience A. Muwanguzi, Robert C. Bollinger, Stuart C. Ray, LaRon E. Nelson, Noah Kiwanuka, José A. Bauermeister, Nelson K. Sewankambo

**Affiliations:** 1 School of Health Sciences, College of Health Sciences, Makerere University, P. O. Box 7072, Kampala, Uganda; 2 Johns Hopkins University School of Medicine, Phipps 540, 600 N. Wolfe St, Baltimore, MD 21286 USA; 3 Johns Hopkins University School of Medicine, 855 N. Wolfe Street room 532, Baltimore, MD 21205-1517 USA; 4 Yale University School of Nursing, 400 West Campus Drive, Orange, CT 06477 USA; 5 University of Pennsylvania School of Nursing, 418 Curie Boulevard, Philadelphia, PA 19104 USA; 6 School of Public Health, College of Health Sciences, Makerere University, P. O. Box 7072, Kampala, Uganda; 7 School of Medicine, College of Health Sciences, Makerere University, P. O. Box 7072, Kampala, Uganda

**Keywords:** Acceptability, Barriers, HIV self-testing, Sub Saharan Africa, Workplace, Men, Qualitative research

## Abstract

**Background:**

Men in Sub-Saharan Africa are less engaged than women in accessing HIV testing and treatment and, consequently, experience higher HIV-related mortality. Reaching men with HIV testing services is challenging, thus, increasing the need for innovative ways to engage men with low access and those at higher risk. In this study, we explore men’s perceptions of drivers and barriers of workplace-based HIV self-testing in Uganda.

**Methods:**

An exploratory study involving men working in private security companies employing more than 50 men in two districts, in central and western Uganda. Focus group discussions and key informant interviews were conducted. Data were analyzed using inductive content analysis.

**Results:**

Forty-eight (48) men from eight private security companies participated in 5 focus group discussions and 17 key informant interviews. Of the 48 men, 14(29.2%) were ages 26–35 years. The majority 31(64.6%) were security guards. The drivers reported for workplace-based HIV self-testing included convenience, autonomy, positive influence from work colleagues, the need for alternative access for HIV testing services, incentives, and involvement of employers. The barriers reported were the prohibitive cost of HIV tests, stigma, lack of testing support, the fear of discrimination and isolation, and concerns around decreased work productivity in the event of a reactive self-test.

**Conclusions:**

We recommend the involvement of employers in workplace-based HIV self-testing to encourage participation by employees. There is need for HIV self-testing support both during and after the testing process. Both employers and employees recommend the use of non-monetary incentives, and regular training about HIV self-testing to increase the uptake and acceptability of HIV testing services at the workplace.

## Background

Globally, men have historically been less likely than women to be tested for HIV and linked to care [1]. Several reasons have been suggested for why men may not agree to HIV testing in sub–Saharan Africa. In Uganda, the working hours of HIV testing services, particularly in health facilities, have been reported as an obstacle to testing [2], while in Burkina Faso, men’s perceived healthy status prevents the uptake of HIV testing [3]. The mobile nature of informal sector employment opportunities for men limits their ability to attend health facilities [4]. Additionally, men have reported that health facilities did not seem to cater to men’s needs [5, 6]. In South Africa, some men view clinics as ‘female spaces’ [7] and HIV testing as a female activity in Lesotho [8]. Men also ‘test by proxy’, believing that the female partners’ results will reflect what their own result would be [9].

In Uganda, 38% of men living with HIV do not know their status [10]. This calls for innovative male-centred approaches to engage men in HIV testing and subsequently link them to care or prevention services [11]. The World Health Organisation recommends HIV self-testing in the workplace as an innovative strategy for reaching men [12]. Workplace-based HIV self-testing involves an employee or employer receiving HIV self-test kits and pre-test support at their work setting, and thereafter taking the test at a convenient and private place such as a private office or at home if preferred. This should be differentiated from home-based HIV self-testing where the test kits are distributed to members in their homes, which provides an opportunity to reach and test couples, children, and families [13]. A few quantitative studies assessing workplace HIV self-testing as a way of engaging men in sub-Saharan Africa have been conducted, however, they have been limited to mining and farming industries in Malawi, Zambia and Zimbabwe [14], as well as truck drivers in Kenya [15]. While these studies reported high acceptability, they did not provide information regarding the facilitators or barriers of this approach, nor provide insights into the approaches to increase uptake of HIV self-testing in work settings. Additionally, the truckers, farmers and miners are largely mobile populations, therefore, there is a gap in information on workplace-based HIV self-testing among men in more stable employment.

In 2019, there were 202 private security companies in Uganda, with about 50,000 security guards employed in those firms [16]. Employees of private security companies represent an ideal population for workplace-based HIV self-testing. They are classified among the priority populations that are currently underserved by HIV testing services [17]. Furthermore, some of their characteristics represent key vulnerabilities for HIV acquisition [12]. Men in security services typically migrate from their homes to work, which places them at high risk of HIV, especially if they remain away from home and/or partners for long periods [18]. They have high alcohol consumption and a relatively low socioeconomic status [19]. Their working hours are arduous, and this sector represents a large population of working men who may not have easy access to HIV testing services [20, 21]. Additionally, HIV testing strategies are typically conducted in public health facilities or government sectors, therefore private security companies provide an avenue of HIV self-testing delivery to the private sector [18].

There is a dearth of literature regarding HIV self-testing initiatives at workplaces in Uganda. Therefore, we conducted this acceptability study before introducing a workplace-based intervention with men in private security companies [22]. The aim of the study was to explore employers and employees’ perceptions of the drivers and barriers of workplace-based HIV self-testing.

## Methods

### Study setting and participants

The exploratory study was conducted between July and September 2019 at private security companies employing at least 50 men in the Hoima and Kampala districts of Uganda. Private security companies in Uganda are typically located in large urban cities. Therefore, Kampala was selected because it has the highest number of private security companies and Hoima district was selected because it is representative of the other urban Ugandan cities. Men were eligible to participate if they were aged 18–60 years and had worked at the company for more than 3 months. Initially, the study team made an appointment and met the owner or the administrative head of each eligible private security company. This meeting granted the team access to the study participants and entry into the company. The team used this preliminary meeting to agree on a suitable day to meet with the employees. While eight private security service companies were involved in the study, the business nature and company policies at three sites did not allow us to hold focus groups there. Therefore, focus groups were conducted at five of the eight venues, and the other three companies provided additional key informants.

### Focus group discussions

Prior to the focus group discussions, the study team provided the employees with information, education, and communication materials on different HIV testing services options. These materials were in the form of posters and leaflets. The posters were placed at strategic locations inside the company and the leaflets were given to individual employees. On a specified date, the study team met the employees in a group at the company premises. This was during the early morning ‘parade’ and the team provided the key aspects of the study. During this meeting, prospective participants watched a 3-min video on the administration of an oral HIV self-test. The study team then invited those who were interested in participating to a private room. The team explained the study in more detail to one employee at a time and obtained written consent prior to enrolment. The enrolled employees were then divided by age; the categories were 18–25, 26–35, 35–45, and 46–60 years. Each group agreed on a time for a focus group discussion at the company premises. The focus group discussions were conducted in a private room, with a minimum of 5 and maximum of 9 men in a group. Each discussion led by a moderator lasted at least 1 h. The data collected included information about HIV testing options and preferences, acceptability, and perceptions of workplace-based HIV self-testing. Participants consented to the presence of a note taker and an audio recording of the focus group discussion.

### Key informant interviews

During the preliminary meeting with the management, the team requested for and obtained a list of all key senior personnel including managers at each company. The study team wrote to each one requesting an appointment. The letter was part of a package which contained information, education, and communication materials about HIV self-testing. On the appointed date, meetings were held in the individual offices of the senior personnel who accepted to meet the team. The team played the 3-min video on oral HIV self-testing. The managers who were willing to participate gave written consent and were either interviewed on the same day or at a later agreed date. The managers had the option of a phone interview or face-to-face interview. The key informant interviews were conducted by the Principal researcher and participants consented to an audio recording of the interview. Each interview lasted 45 min to 1 h and employed an interview guide.

Data collected included participants perceptions of HIV testing options, acceptability of workplace-based HIV self-testing, perceptions about the staff getting tested at the company premises, concerns, and what they could do to facilitate HIV self-testing at the company. The participant sample size was guided by data saturation, whereby recruitment stopped when no new information arose from participant interaction.

### Data analysis

The key informant interviews and focus group discussions audio recordings and field notes were transcribed verbatim and analysed manually using qualitative inductive content analysis [23]. Initially two team members (PAM and RN) reviewed the transcripts while listening to the audio recordings to ensure that all the information was captured. The pair undertook the coding process separately to identify meaningful phrases and then came together to agree on the identified codes. Any disagreements on the codes were settled by a third member of the study team. The coding team placed the codes into groups according to their similarity of meaning to form subcategories and then categories. The categories that contained similar meanings were further grouped into emergent themes. Finally, a sample of the study participants reviewed the categories and themes for credibility of the data analysis. Any disagreements that arose were resolved by revisiting the verbatim transcripts, editing the categories and themes for the correct meaning and re-checking with the participants until consensus was achieved.

## Results

### Participants characteristics

Forty-eight (48) men from eight private security companies participated in 5 focus group discussions (*n* = 31) and key informant interviews (*n* = 17). Of the 48 men, 14(29.2%) were ages 26–35 years, and the majority 31(64.6%) were security guards. Seventeen (35.4%) of the participants did not know their current HIV status (Table 1).

**Table 1:** Demographic characteristics of men in private security services *N* = 48

Participant Characteristics	Frequency (percentage %)
**Age range, years**	
18–25	12 (25.0)
26–35	14 (29.2)
36–45	11 (22.9)
46–60	11 (22.9)
**Relationship status**	
Married/Cohabiting	23 (47.9)
Unmarried	17 (35.4)
Divorced/separated	8 (16.7)
**HIV status (self-reported)**	
Known	31 (64.6)
Unknown	17 (35.4)
**Employment Category**	
Company Owner	2 (4.2)
Senior Management	6 (12.5)
Middle Management	9 (18.8)
Security guard/ employee	31 (64.6)

### Drivers and barriers to workplace-based HIV self-testing

**Fig. 1:**
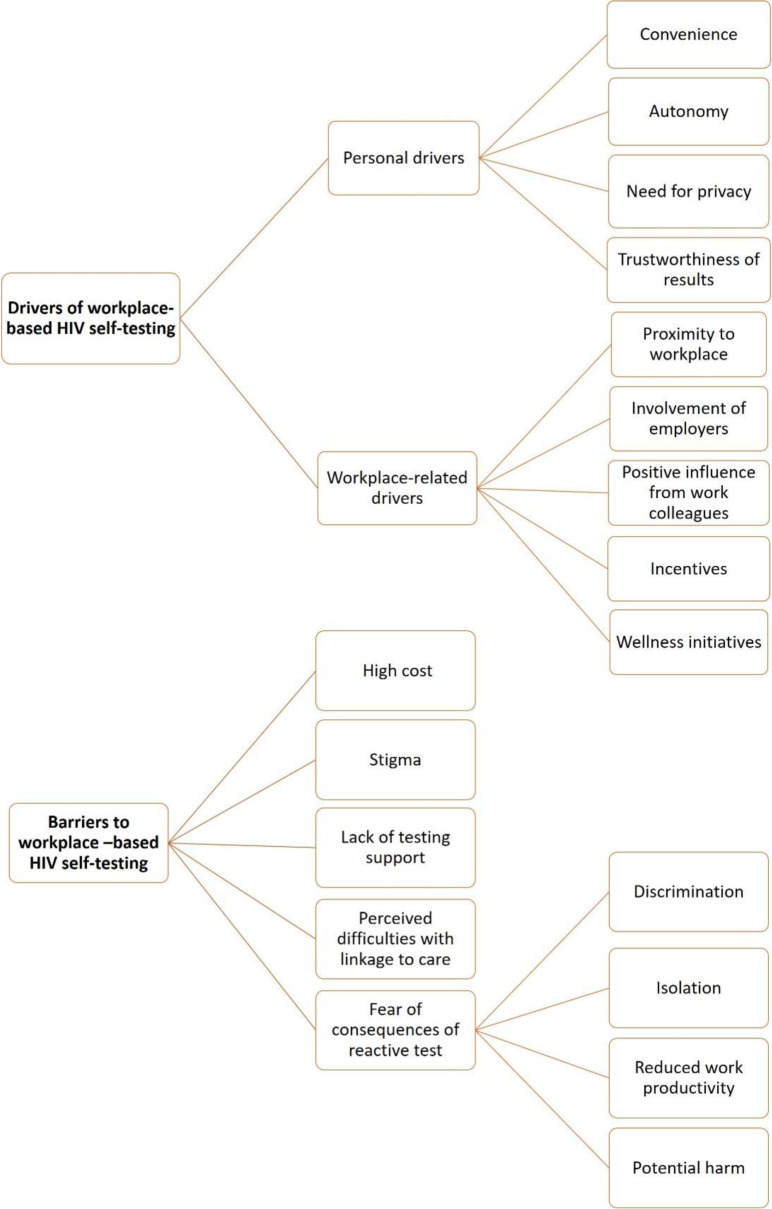
Summary of categories and subcategories for driver and barriers to workplace HIV self-testing

To elicit these responses, the participants responded to questions about the potential barriers to workplace-based HIV self-testing. The questions included *What are the advantages of taking an HIV test at the workplace? What are the disadvantages? Would you consider taking an HIV self-test at your work premises? What would motivate the decision to take the HIV self-test? Do you foresee any potential challenges of HIV self-testing at the workplace?* A summary of the categories and subcategories is shown in Fig. 1.

### Drivers of workplace-based HIV self-testing HIV self-testing

The Coding tree of the potential drivers and barriers to workplace-based HIV self-testing among men is presented in Table 2.

**Table 2:** Coding tree of the potential drivers and barriers to workplace-based HIV self-testing among men

Theme	Category	Sub-category	Codes	No of participants	
				Focus group discussions ***n*** = 31	Key informant interviews ***n*** = 17
**Drivers of workplace-based HIV self-testing**	Convenience		No days off, No extra expenditure on transport, No lines at hospital, Don’t have to go to hospital, Finds me here, Information provided, Don’t have to miss work, No loss of production, Freely available, No pain	22	8
	Autonomy		Can decide for myself, My choice, Don’t feel pressured, Decide when and how to test, It is up to me	20	11
	Proximity of testing services		My hours are demanding, Never have time, Office testing can be managed	19	8
	Incentives		Don’t give money, Make promotions, Raffles, Give us small encouragement	16	0
	trustworthiness of HIV self-testing		Do it myself, My results are mine, No mistakes, Trust my eyes	16	3
	Need for privacy		Know my status alone, No one will see, No one knows, Bosses will not know	20	9
	Combined Health Initiatives		Hide it in other tests, Day for all tests, Test also for syphilis and prostate	15	17
	Employer involvement		Bosses test with us, Employers example, Health awareness information sessions, Allow health programs at workplace	17	3
	Influence from work colleagues		Friends at work, Workmates, Fellow workers	7	0
**Barriers of workplace-based HIV self-testing**	Stigma		People will see me picking the kit, people will suspect, colleagues will see, people monitor my mood, how to hide the used kit	15	3
	Fear of the consequences of a reactive self-test	Discrimination	Too much work or, too little work assigned	18	4
		Decreased work productivity	Absenteeism, Depressed, may affect my work, Demoralize employees, Won’t work as hard, Need time off	3	13
		Isolation	Lose friends at work, People fear you, Telling friends and family	12	0
	Concern regarding unassisted HIV self-testing		Someone to talk to, interpreting test results, Guidance on the kit, how to use the kit, how to perform the test	8	9
	Cost of HIV self-test kits		Too expensive, It’s not free, Not free, Could pay if discounted, Money is a lot, Not affordable, Can pay little	25	15
	Perceived difficulty in linkage to confirmatory testing		Where to go? who to receive me? List of hospitals, Clinics nearby expensive, no time, Government facility far, what to do after testing, ARVs for life, manageable?	13	3

#### Convenience

Some employees 22/31 and key informants 8/17 agreed that one of the reasons they would consider taking an HIV self-testing at the workplace is because of convenience (Table 2). The participants felt that HIV self-testing was more agreeable because it could simply be picked up from the workplace with minimal fuss. Additionally, the oral HIV self-testing kit was more favourable since it did not involve a needle prick. Below is an excerpt from a respondent,

*It will make my work easy, for example if I am going to report on duty, I do not have to worry about spending time at the hospital, I will get the kit at work and test at my own time. -*Focus group discussion 5, participant 02, 49 years

#### Autonomy

The men liked the sense of freedom that comes with choice. They reported that having the test kit gave them the power to choose when and where to take the test. They felt that they could take the test when they felt physically and psychologically ready to know the results. Additionally, they could decide whether to use the self-test kit or not as evidenced in the excerpt below,

*It allows me not to feel pressured to take the test because the employers have said so. This test kit gives me the freedom of choice.* - Focus group discussion 1, participant 06, 24 years

#### Need for privacy

The men felt that the workplace was a good place to take an HIV self-test and then get the results privately. Although they received the test kit at work, they did not have to take the test there, they could do it privately at home, or any other place where they felt safe. This is expressed in the excerpt below,

*It helps me to know my status, it is personal and after knowing my results, I don’t have to tell anyone what my results are or that I have taken a test…..you see your results alone* - Focus group discussion 2, participant 03, 28 years

#### Trustworthiness of testing process and results

The men did not completely trust the health facilities where they usually access HIV testing services. They reported that sometimes health workers give wrong results and there was no way of knowing if those were one’s true results. They felt that if they took the HIV self-test at the workplace, the results would be more accurate since they were taking their own readings. The excerpt below highlights a representative opinion of several employees.

*Sometimes in the health centre they may tell you that you are positive when you are negative or vice versa because they test you and just give you results but for HIV self-test at work, you are very sure of your status because you are doing it yourself*. - Focus group discussion 4, participant 05, 30 years

#### Proximity of HIV testing services

The demanding nature of work and unusual working hours were cited as a major barrier to facility-based HIV testing services and thus a strong driver for HIV self-testing at the workplace. The men felt that they were more likely to take an HIV self-test because it was available at the workplace, which they would not ordinarily do because it would entail traveling to the health facility. One respondent spoke about this,

*there is no time to go for testing, in addition most times our schedules clash with the timetable for the outreaches in the community which does not allow us enough time to get there.* - Focus group discussion 3, participant 01, 40 years

#### Involvement of employers

The men expressed that they would be more willing to take an HIV self-test if they saw their supervisors and employers also actively engaged. They were concerned about taking part in initiatives where the employers did not participate. The main worries were around their job safety, and the possible repercussions of returning positive or reactive test results.

*The bosses should also join us in testing to make it clear that it is ok. They should also make it clear that if we are tested positive, they will allow us to go for treatment and no one will get fired for being HIV positive.* - Focus group discussion 3, participant 03, 37 years

The key decision makers were willing to get involved in workplace initiatives if it was more likely to motivate the employees engaged. However, this was on condition that they do not share their test results. This is expressed below,

***Key informant interview question: The team proposed that the company owners and senior personnel should participate actively in workplace initiatives. What is your opinion on this?***

*I am willing to participate in this endeavour if they do not want to see my test results. I can come and join them and even pick up the test kit, whether I use it is another matter. But if it will make them feel more comfortable to get involved then I can do it.* Key informant 04, 51 years

#### Positive influence from work colleagues

Peer influence at the workplace was a strong potential motivator for taking HIV self-testing. The men reported that they were likely to take an HIV self-test at the workplace if they were persuaded, of if they saw their colleagues taking the test. This is illustrated in the quote below,

*For example, a team from the hospital came here to talk about circumcision and it was something I had never thought I could do. But after the meeting, one of my colleagues asked me to go with him to the facility. I ended up getting the operation* [Voluntary Male Medical Circumcision] *and later persuaded two other work colleagues to do the same*. - Focus group discussion 2, participant 04, 30 years

#### Incentives

The participants proposed that employers might consider incentivizing their employees to take an HIV self-test. They said they would get motivated if they had some meaningful non-cash incentives. These ranged from t-shirts to days off in some cases. While the employees thought it was a great idea, the employers were less enthusiastic about incentivizing employees to take an HIV self-test. They worried that it might be too costly and thus unsustainable. They also thought that the greatest motivator should be the employees desire for better health, rather than incentives.

*My proposal is that they give some things to make people to test. For example, if you take a test, you can get phone airtime, t-shirts, one or two days off or lunch or somethings like that.* - Focus group discussion 4, participant 01, 34 years

*I think one of the most important things that we can be given is education. During the training they can include some items on HIV prevention, testing and treatment.* - Focus group discussion 4, Participant 06, 29 years

Key informant interview question: some of the men are proposing that we give some incentives to encourage them to take HIV self-testing. What is your opinion on this?

*Hmmm…. while I can see the value in what they propose, it comes at a great cost to the company. No, I do not think it is feasible, they should know it is for their own good not because of gifts. -*Key informant 15, 47 years.

#### Wellness days and combination health initiatives

The men were enthused by the possibility of taking HIV self-testing at the workplace because it eliminated the hurdles, they usually face with facility-based HIV testing services. Several men (32/48) requested that the HIV self-testing be offered as part of combined health initiatives at the workplace. They proposed the introduction of an annual health and wellness event where HIV self-testing could be offered in combination with other tests like those for Sexually transmitted infections, Diabetes Mellitus, Hypertension, prostate cancer, and assessments like Body Mass Index, dental and other activities so that that the HIV test can be viewed as any other assessment. This idea was voiced by many of the employees as shown in the excerpt below,

*I think people fear to come directly for the HIV test here at the office, but if you bring different tests here, someone can easily quietly receive the test kits. We can receive the counselling together and then we go for different tests like for checking the prostate or syphilis. -* Focus group discussion 2, participant 02, 27 years

All key decision makers were willing to have the wellness events at their premises if it was either free or affordable for the company and did not interfere with the employees’ work schedules. While they wanted to improve the health and wellness of their employees, they were not willing to expend resources on costly wellness interventions, or initiatives that would take the men away from their work.

***Key informant interview question: The employees propose a free annual health and wellness day with several tests and assessments including HIV self-testing at the company premises. What are your thoughts about this?***

*Oh…that is a good idea which sounds expensive. We would be happy to partner with organizations that can offer these services free of charge or at a very minimal fee and it should not happen when they are supposed to be working. When our employees are healthy, it means they will be more productive with less time off due to improvement in health status. -*Key informant 04, 51 years

### Barriers to workplace-based HIV self-testing

#### High cost of HIV self-test kit

One of the major barriers to HIV self-testing was the prohibitive cost of the test kit (40/48). The men were surprised that the HIV self-test kit was not free. The test kit costs approximately $4–5 on the open Ugandan market. They suggested that it should be free like the HIV testing service offered at the health facility. Some felt that in that case, it was still much cheaper to pay the cost of transport to go and take a free test at a health facility. One participant shared,

*It will still be cheaper for me to go to the hospital because the transport fare is not that much and the test at the hospital is free.* - Focus group discussion 1, participant 03, 22 years

The employers were sceptical and categorically expressed their concern about the high cost of the test kits. For example, one senior manager thought this was too expensive. Below is an excerpt from the interview:

*If one employs 2000 security guards’ and about 30 administration and management staff. This will cost over 30 million Uganda shillings!* [$8000] *Maybe the government should consider subsidizing or giving them out for free otherwise it might not work. -*Key informant *17*, 49 years

#### Stigma

The men felt that just picking up an HIV self-test kit would invite unwanted scrutiny from their work colleagues. Several men suggested that people typically take an HIV test because of high-risk behaviour or because they were uncertain of their HIV status. Therefore, they were worried that if their work colleagues saw them picking an HIV test kit, they might conclude that they are involved in risky sexual behaviour. They felt that this was a barrier to taking an HIV self-test at the workplace. Additionally, they felt that their work colleagues would watch them closely for any change in their mood following their tests, which may lead to unintentional disclosure. Several respondents verbalized sentiments such as the one below:

*Once people see you picking the Kit, they* [work colleagues] *will observe you for long to see whether you are sad or happy since the kit is strictly for HIV. This is different from the hospital where you can go and get tested for many different diseases.* -Focus group discussion 3, participant 05, 36 years

The men were also concerned about how to dispose of the HIV self-test kit after taking the test. They were worried that if a co-worker found the kit, they might work out who it belongs to and disclose this individual’s status. This is expressed below,

*Another disadvantage is discarding of the HIV self-test kit, unlike in the hospital where the health workers keep the kits, if you throw this one away at home anyhow or at work, other members who find it can easily know your status.* -Focus group discussion 2, participant 03, 31 years

#### Lack of testing support

Some of the men felt that they needed someone to talk to following the self-test. They expressed that the person should be someone who adheres to the principle of confidentiality. However, they requested that it should be someone away from the work premises, who can remain neutral. One participant shared,

*… provide a number for someone I can call, so that whatever the result, I would want someone to talk to who I know will not tell people. In case the number is not provided, you find a hard time knowing who to talk to.*-Focus group discussion 3, participant 07, 39 years

One senior officer suggested the inclusion of some form of assisted HIV self-testing.

*In the health centre at least, you have some counselling before you receive a test. I know you have explained that they will receive information in the test kit but that is not enough. Could we have someone here to at least explain to them how the test works, where to go for more services and to be available afterwards?* -Key informant 06, 42 years

Several participants were concerned about the additional cost of hiring a counsellor or of creating additional space for such activities, because they felt that some form of privacy was required for these activities.

*We barely have enough money to pay our staff, who is going to pay for a counsellor? Additionally, where will this be done? We do not have a clinic so that means we must make space for this person. That could be a problem. -Key informant 17*, 49 years

#### Perceived challenges in linking to confirmatory testing

Some of the men were concerned about the transition from testing at the workplace to receiving post-test services at health facilities. They had prior bad experience at the health facility and felt that it might be better to take the test at a health facility because one was already in the system. They worried about taking a test out of the facility and then seeking treatment from the facility. They wanted a reassurance, that this transition had been considered and would be seamless. This is illustrated below,

*…. However, I fear that if I am found to be positive, it will be difficult for me to access services at the hospital. It may just be easier for me to go to the hospital at once instead of testing at the workplace.* - Focus group discussion 1, participant 05, 18 years

### Fear of the consequences of a reactive self-test

#### Discrimination

The employees were cautious about taking an HIV self-test at the workplace because they were worried about the penalty of receiving a positive test result. The men believed that their supervisors would discriminate against them if they got a reactive self-test. This respondent verbalized that they did not want to be viewed differently from their peers.

*they will either give you too much work so they can get rid of you, or they will give you too little work thinking that you are going to die tomorrow. You will not get any overtime or extra work shifts which may result in a reduced income.*- Focus group discussion 5, participant 06, 47 years

As part of the interviews with key senior personnel, we asked if they would treat their employees any differently if they knew their HIV status. While some of them would treat their employees the same as everyone else, others felt that they would treat them differently. They thought that it might even be done unintentionally.

***Key informant interview question: How would you treat the employees if you knew their HIV status?***

*If I found out that someone was positive, I would try to treat them equally with their colleagues. HIV is not like other diseases, if they take their medicine, I think they can work normally.* - Key informant 12, 40 years

On the other hand, another senior manager alluded to treating an employee differently because of their HIV status. This validates the employees concerns regarding the potential negative repercussions of receiving a reactive test result. He shared,

*Honestly being human, the moment you get to know someone’s HIV status, you see them in a different light. I would not want to put them on a night shift or give them strenuous work or double shifts if they are sick* [test positive for HIV]*. I do not want to be responsible for making someone’s condition worse.* -Key informant 05, 43 years

#### Isolation

Friendships and relationships were important to the men and they worried that they would be isolated by their co-workers if they received a reactive self-test. They were not sure how their colleagues would view them if they received a positive result. This was important because they shared spaces like locker rooms and were troubled about being isolated from friends at the workplace. This was expressed by several men as this excerpt shows,

*Some fear to go because once people know you have it, they will isolate you and they will not allow to get deployed with you.* - Focus group discussion 5, participant 02, 49 years

#### Decreased work productivity

The men were unwilling to take HIV self-testing at the workplace because they worried that they might not be able to do their duty as well in the event of a reactive self-test. This was more about being psychologically unable to perform their work because they might nor focus or concentrate on their jobs. One participant narrated,

*You will spend so much time thinking about the HIV results and this may make you unproductive at work. You lose of interest in working because you imagine that you are going to die tomorrow. -* Focus group discussion 1, participant 03, 25 years

Prompt; How different is this from receiving unexpected results at the health facility?

*Usually, you go to the health centre for a test when you are feeling sick, but here I am not sick and then I take the test and find that I am positive* [receive a reactive self-test], *how can I be able to work properly after that? It will make me depressed and affect my work.* -Focus group discussion 1, participant 03, 25 years

Employer alluded to consideration of the return on investment. He suggested that if the testing is done at the workplace, the employers feel a certain sense of responsibility for an employee who tests positive. This meant that the company would have to give time away from work for clinic visits, which he felt was not fair. He shared,

*Some may end up going into shock and may fail to come to work and with our type of work even a single missed shift is a poor return on investment.* - Key informant 01, 55 years

#### Potential for harm

Some of the men were concerned about the potential to harm themselves or their partners in the event of a reactive-self test. They felt that they were at a low risk for HIV acquisition, which could only mean that their partner was the source of the infection. They worried about the likelihood of committing intimate partner violence. This was one of the reasons why they were unlikely to take an HIV self-test at the workplace.

*If I know myself and I am sure my life has been ok, I will start thinking about my family, whether I was born with HIV or it would be my partner and I would go home and force my partner to test so that we know.* -Key informant 08, 37 years

## Discussion

Workplace-based HIV self-testing may help in reaching a vulnerable population of high-risk men. In this study, men highlighted several potential drivers for workplace-based HIV self-testing, including convenience, autonomy, the need for privacy, the need for alternative means of accessing HIV testing services, personal beliefs about the trustworthiness of HIV self-test results, wellness events, incentives, influence from work colleagues and the involvement of the employers. However, men also recognized several barriers, such as the high cost of the test kits, stigma, fear of the consequences of a reactive self-test result and the lack of post-test support. Given the acceptability of HIV self-testing in the workplace among men interviewed, efforts to curtail the barriers are needed. In the discussion, we explore the drivers and barriers that have not been widely discussed in the literature.

Employer involvement is key to the realization of any HIV initiative in the work setting and will only succeed with the participation of people at all levels of the organization [24, 25]. In this study, the employees were keen for the employers to participate in HIV self-testing, they felt that this would give them confidence and positively influence their participation. On the contrary, the employers were more sceptical about HIV self-testing because of the high cost of the test kits and non-monetary incentives. Additionally, the need to rearrange existing spaces and hire a counsellor to offer post-test support was not welcome for most of them. The employers were also not keen to have HIV self-testing initiatives offered during working hours because that meant reduced productivity from their employees or the need to provide paid time off for clinic visits. This highlights the need to involve employers at every stage of planning, as they can either be the biggest motivator or present a barrier to workplace HIV self-testing initiatives.

Men in this study felt that the HIV self-test results were more trustworthy than those received during standard HIV testing services at health facilities. This finding is consistent with Choko and colleagues who reported that 81 out of 88 (92.1%) fishermen in Uganda trusted the HIV self-testing results [26]. Given different self-testing assays, it will be important to ascertain whether different groups of men prefer HIV self-testing through blood as compared to oral sampling. For example, a study in Botswana reported that participants were willing to use HIV self-test kits, particularly if the kit utilized blood specimens given the perception that these tests were more trustworthy [27]. This suggests the need for sensitization campaigns prior to the utilization of the test kits at the workplace and to demystify peoples’ assumptions regarding the trustworthiness of test sampling method.

Work demands and lack of time have been consistently reported as reasons for men’s non-engagement in HIV testing services [4]. This was no different in this study where the proximity to HIV testing services was a driver for workplace-based HIV self-testing. Workplace-based HIV self-testing is a possible strategy that may help to reduce the current facility-based testing challenges, including long lines, long waiting periods and not enough counsellors [2, 12, 28]. Unfortunately, the challenge of linkage to care following HIV self-testing still persists [29]. Oduetse and colleagues propose follow-up support for all those who collect test kits, which would help improve linkage to posttest services [27]. The men in the study suggested the need for post-test support including the presence of a counsellor who can provide further information on the next steps. The literature continually highlights this as a major challenge for HIV self-testing. There is need to develop and test interventions to improve linkage to HIV prevention or care following HIV self-testing at the workplace.

Men reported stigma as a major potential barrier to workplace-based HIV self-testing, including concerns about being stigmatized for simply picking up the test kit. This resonates with findings from a study in Eastern Uganda, where men expressed concern about accessing non-facility-based HIV testing due to informal monitoring and people watching to see who was taking the test [4]. Similarly, participants in Botswana felt that the kits should be distributed strictly at health facilities to prevent stigma [27]. Nonetheless, the men felt that HIV self-testing afforded them privacy while testing and the confidentiality of their results especially from their employers and peers. Studies have shown that men prefer HIV self-testing because it offers more privacy and confidentiality [30, 31]. The men in the study also proposed the introduction of wellness days to incorporate several other health assessments to reduce stigma and improve the uptake of workplace-based HIV self-testing [32, 33]. The barriers highlighted here, make a case for home-based HIV self-testing which may address some of the men’s concerns such as disposal of the HIV self-test kits, unintentional disclosure to the workmates, and colleague’s discernment of one’s mood following a positive test result. Choko and colleagues, [34] reported that community members considered home-based HIV self-testing acceptable, if they did not have to divulge their results to the people distributing the kits. On the other hand, work-place based HIV self-testing has the advantage of reaching men where they are in the daytime. Therefore, researchers or programs that plan to conduct HIV self-testing initiatives at the workplace should pre-plan for mitigation measures to address these valid concerns. Additionally, participants can be encouraged to receive the kit at work, and administer the test at home, in private.

Another major potential barrier to workplace-based HIV self-testing is the cost of the test kits. Harichund and colleagues refer to the cost and accessibility of test kits as the first barrier to preventing HIV status awareness [35]. In this study, both the employers and employees were concerned about the cost of the kits being too high for individuals and the company, respectively. While the cost may not be exorbitant, it may be quite high for most at risk populations and the poor and may unfortunately not reach those that need the testing the most [36, 37]. However, in Tanzania, men regarded the benefits of HIV self-testing over costs as savings made on the money paid to test in facilities and private clinics and on follow-up fees and time saved in other income generating activities [38]. This makes HIV self-testing convenient [31]. The men in this study proposed that the cost of the kits should be reduced or that they should be provided freely like other HIV testing modalities. This agrees with other proposals for financial discounts [36] or even free self-testing kits to those people who have low income and high risk of HIV [38].

Participants in this study were concerned by the limited support offered for HIV self-testing beyond the pre-test counselling. There have been mixed reactions in the literature to the lack of face-to-face counselling. In some studies, participants preferred HIV self-testing because they did not have to have face-to-face counselling [30]. In contrast, other studies showed that some people were unlikely to take an HIV self-test due to the absence of counselling [30]. Some people preferred a standard test because they were worried about coping with the results on their own and the failure to link to posttest services [37, 42]. Additionally, policy makers, for example expressed concern that the lack of face-to-face support may increase the risk of psychopathic tendencies and suicidal ideation and coercion [39, 40]. In that regard, Youngs and colleagues contend that it is unlikely that the short posttest counselling offered is enough to mitigate the potential psychological effects of a reactive self-test [36]. Thus, the recommendation is to offer ongoing posttest support rather than to simply offer posttest counselling. Additionally, although first-time testers felt confident enough to perform an unsupervised HIV self-test, they would have welcomed support during the testing process [31]. This could include support in areas such as interpreting the test results [15]. Additionally, there should be mechanisms in place for people to ask questions at each step of the self-test. These might include telephone hotlines, mobile phone text messages, videos, social media, and Internet-based applications to provide technical support, counselling and referrals for further HIV testing services, HIV prevention, care and treatment and other services [15, 29].

### Study limitations

The method of focus group discussions may have led to a social desirability bias given the nature of the topic. Additionally, some critical information may have been lost because employees only participated in focus group discussions and did not have the opportunity to participate in an individual interview.

### Study strengths

This study identified the drivers and barriers of HIV self-testing among high-risk men at workplaces in Uganda. This study also highlights the perspectives of the employers, key senior personnel, and employees. The innovative participant triangulation method of involving these different stakeholders is central to the future success of workplace-based HIV self-testing initiatives, since they provide us with a holistic picture. Future quantitative studies in Uganda or the region aimed at developing workplace-based HIV self-testing interventions may utilize the findings of this study to guide the design of these initiatives.

## Conclusion

We make the following recommendations based on our study findings. First, we suggest the involvement of the employers at every stage of the workplace-based HIV self-testing initiatives, including planning and implementation. This will give the workers more confidence to participate as well. Second, we recommend some form of HIV self-testing support both during and after the testing process. This may be a toll-free hotline, peer support, assistance in interpreting results or a counsellor available to answer any questions, especially for first-time testers. Thirdly, we recommend the introduction of regular wellness initiatives alongside the HIV self-testing services. These wellness initiatives may involve various health promotion activities alongside HIV self-testing, including blood pressure and blood glucose measurement, sexually transmitted infections and prostate cancer screening and health talks, among others. Fourth, to increase uptake of HIV self-testing at men’s workplaces, we propose the use of simple non-monetary incentives. Finally, both employers and employees recommended regular sensitization and training regarding HIV self-testing, to increase the uptake and acceptability of testing at the workplace.

## Data Availability

The datasets used and/or analysed during the current study are available from the corresponding author on reasonable request.
